# Rewriting the MASLD‐associated hepatocellular carcinoma script: Targeting epigenetics and metabolism

**DOI:** 10.1002/ijc.70047

**Published:** 2025-07-22

**Authors:** Chiara Aiello, Eric Felli, Teresa Musarra, Lorenzo Nevi, Annamaria Altomare, Jordi Gracia‐Sancho, Andrea Baiocchini, Simone Carotti

**Affiliations:** ^1^ Microscopic and Ultrastructural Anatomy Research Unit, Department of Medicine and Surgery Università Campus Bio‐Medico di Roma Rome Italy; ^2^ Department of Visceral Surgery and Medicine Inselspital, Bern University Hospital, University of Bern Bern Switzerland; ^3^ Department for BioMedical Research, Visceral Surgery and Medicine University of Bern Bern Switzerland; ^4^ Department of Pathology Ospedale San Camillo Forlanini Rome Italy; ^5^ Departmental Faculty of Sciences and Technology for Sustainable Development and One Health Università Campus Biomedico di Roma Rome Italy; ^6^ Liver Vascular Biology IDIBAPS Biomedical Research Institute, CIBEREHD Barcelona Spain; ^7^ Predictive Molecular Diagnostics Fondazione Policlinico Universitario Campus Bio‐Medico Rome Italy

**Keywords:** chronic liver disease, epigenetic mechanisms, HCC, MASLD

## Abstract

Abnormal epigenetic patterns are crucial for the progression of metabolic‐associated steatotic liver disease (MASLD) and hepatocellular carcinoma (HCC) development. Recent research focused on the interplay between epigenetics and altered metabolic pathways in HCC from MASLD, suggesting that the reversibility of epigenetic changes offers potential for new therapeutic strategies. However, identifying effective epigenetic targets remains challenging due to the diverse etiologies of HCC. Targeting metabolic reprogramming, MASLD‐associated HCC shows promise in reducing lipid anabolism. In this context, a more comprehensive understanding of metabolomic changes closely tied to druggable epigenetic modifications is of utmost importance. This review provides a concise summary of the key factors driving hepatocarcinogenesis in MASLD and delves deeper into the epigenetic mechanisms. Finally, we aim to present an overview of the primary mechanisms concerning the intricate bidirectional interplay between the metabolome and epigenetic modifications in MASLD‐associated HCC, along with therapeutic strategies specifically targeting epigenetics and metabolomic alterations.

## Graphical abstract

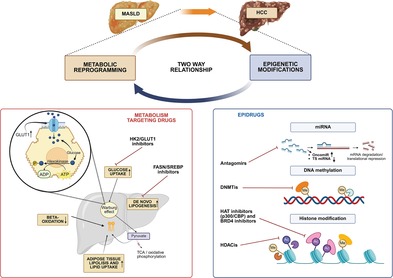

**Abbreviations**
ACC: acetyl‐CoA carboxylase
ACLY: ATP citrate lyase
ACSL4: acyl‐coenzyme A synthetase long chain family member 4
ALKBH5: AlkB homolog 5
BAs: bile acids
Bmf: Bcl2‐modifying factor
BRD4: bromodomain and extra terminal domain 4
CASC2: cancer susceptibility candidate 2
C/EBP‐β: CCAAT enhancer binding protein beta
ChREBP: carbohydrate‐responsive element‐binding protein
circRNAs: circular RNAs
CSC: cancer stem cell
CTNNB1: catenin beta 1
DACT2: disheveled binding antagonist of beta catenin 2
DCAF4L2: DDB1 and CUL4‐associated factor 4 like 2
DM2: type 2 diabetes mellitus
DNL: de novo lipogenesis
DNMTis: DNA methyltransferases inhibitors
DNMTs: DNA methyltransferases
ER: endoplasmic reticulum
EZH2: enhancer of zeste homolog 2
FASN: fatty acid synthase
FATPs: fatty acid transport proteins
GLUT1: the glucose transporter 1
GNMT: glycine‐N‐methyltransferase
GPX: glutathione peroxidase
GSH: glutathione
H3Q5ser: histone H3 glutamine 5 serotonylation
HATs: acetyltransferases
HCC: hepatocellular carcinoma
HDACis: deacetylases inhibitors
HDACs: deacetylases
HKs: hexokinases
HMTs: histone methyltransferases
HPTMs: histone post‐translational modifications
hTERT: human telomerase reverse transcriptase
IL: interleukin
IRS‐1/2: insulin receptor substrate‐1/2
KDMs: histone lysine demethylases
KLF5: Kruppel‐like factor 5
LEFTY2: left–right determination factor 2
lncRNAs: long non‐coding RNAs
LPLs: lipoprotein lipase
m6A: N6‐methyladenosine
MAPK: mitogen‐activated protein kinase
MASH: metabolic‐associated steatohepatitis
MASLD: metabolic‐associated steatotic liver disease
MAT1A: methionine adenosyltransferase 1A
MBD2: methyl‐CpG binding domain protein 2
ME1: NADP‐dependent malic enzyme
METTL3: methyltransferase 3,N6‐adenosine‐methyltransferase complex catalytic subunit
miRNAs: microRNAs
mTOR: mammalian target of rapamycin
MTTP: microsomal triglyceride transfer protein gene
ncRNAs: non‐coding RNAs
NEAT1: nuclear enriched abundant transcript 1
NF‐κB: nuclear factor kappa B subunit 1
NK: natural killer
NOTCH3: neurogenic locus notch homolog protein 3
PD‐1: programmed cell death protein 1
PGAM5: deacetylates phosphoglycerate mutase 5
PI‐3 K: phosphoinositide 3‐kinase
PKM2: pyruvate kinase M1/2
PNPLA3: patatin‐like phospholipase domain‐containing 3
PPARGC1‐A: PPARG coactivator 1 alpha
PTEN: phosphatase and tensin homolog
PUFA‐OOH: polyunsaturated fatty acids peroxides
RASSF1: Ras association domain family member 1
ROS: reactive oxygen species
SAM: S‐adenosylmethionine
SCD1: stearoyl‐CoA desaturase 1
SCFAs: short‐chain fatty acids
SIRTs: sirtuins
SLC7A11: solute carrier family 7 member 11
SNPs: single nucleotide polymorphisms
SOCS1: suppressors of cytokine signaling 1
SREBP‐1: sterol regulatory element‐binding protein 1
TGM2: transglutaminase 2
TM6SF2: transmembrane 6 superfamily member 2
TME: tumor microenvironment
TP53: tumor protein p53
TSG: tumor suppressor gene
TUBA1B: tubulin alpha 1b
UPR: unfolded protein response
VEGF: vascular endothelial growth factor
VLDL: very low‐density lipoprotein

## 1 INTRODUCTION

Metabolic‐associated steatotic liver disease (MASLD)‐associated hepatocellular carcinoma (HCC) represents a rapidly growing global health concern.^1^ Chronic liver injuries resulting from MASLD and metabolic‐associated steatohepatitis (MASH) are increasing causes of primary HCC.^2^ Among primary liver malignancies, HCC represents 75%–85%^3^ and is ranked as the fourth most prevalent cancer globally.^4^ Key risk factors for the progression of MASLD to HCC, even in the absence of cirrhosis, include older age and the presence of metabolic disease like diabetes.^5^, ^6^

Epigenetics has emerged as a key area of study in understanding MASLD and HCC development.^7^ An important feature of MASLD‐HCC is its ability to promote lipid accumulation, glycolysis, and fatty acid synthesis, which collectively define a process known as metabolic reprogramming.^8^ This adaptation is critical to meet the biosynthetic demands of tumor growth.^9^ As previously described, epigenetic changes and metabolic reprogramming are deeply interconnected in MASLD‐HCC progression.^10^ While the emerging evidence on the epigenetic scars underlying the progression to HCC opens new horizons, their clinical translation is still limited. This article review aims at bridging the different experimental findings to support a comprehensive knowledge of this field.

## 2 MAIN DRIVING MECHANISMS FROM MASLD TO HCC

In MASLD, main drivers for the development of HCC are insulin resistance, lipotoxicity, oxidative DNA damage, and immune‐related mechanisms. Additional factors, such as genetic predisposition, gut dysbiosis, hepatic biomechanical alterations, and the use of alcohol and tobacco, can further influence the risk.^11^ Impaired insulin sensitivity is responsible for cell proliferation and blocks cell death, promoting liver cancer development. Frequent blood glucose peaks activate the insulin receptor substrate‐1/2 (IRS‐1/2) pathway through phosphoinositide 3‐kinase (PI‐3 K) and mitogen‐activated protein kinase (MAPK).^12^, ^13^ High plasma insulin levels stimulate fat production via the rapamycin complex‐1/ Sterol regulatory element‐binding protein 1 (SREBP‐1) axis and angiogenesis through vascular endothelial growth factor (VEGF) expression.^14^, ^15^

Lipotoxicity, via lipid overload in hepatocytes, overcomes antioxidant defenses, sustains chronic inflammation, and causes cell death via lipid oxidation and reactive oxygen species (ROS) production.^16^ Primary mechanisms are the stress of the endoplasmic reticulum (ER), mitochondrial damage, ER‐mitochondrial contacts, and lysosomal disruption, with consequent non‐apoptotic cell death.^17^, ^18^ The ER, responsible for lipid production and protein folding, activates the unfolded protein response (UPR), a driver of inflammation, cell death, and fibrosis. Interestingly, besides lipid overload, high levels of fructose in the liver can trigger ER stress via activation of NF‐kB and Janus kinase.^19^ Lipotoxicity and ROS also induce ferroptosis, thanks to polyunsaturated fatty acids peroxides (PUFA‐OOH).^20^ Ferroptosis plays an important role in sorafenib resistance in HCC, mediated by SLC27A5 downregulation.^21^

These metabolic processes are intricately regulated and interdependent. For instance, insulin resistance drives hepatic lipid accumulation but also exacerbates oxidative stress and inflammation, creating a feedback loop that promotes both steatohepatitis and tumorigenesis. Moreover, the interplay between metabolic dysregulation and immune responses, as well as the influence of the gut‐liver axis, further adds layers of complexity to MASLD‐HCC pathogenesis.

## 3 GENETIC AND MOLECULAR RISK MODIFIERS IN MASLD‐ASSOCIATED HCC

Gene modifiers like specific single nucleotide polymorphisms (SNPs), such as patatin‐like phospholipase domain‐containing 3 (PNPLA3) and transmembrane 6 superfamily member 2 (TM6SF2), impact MASLD severity, increasing the risk of complications like HCC.^22^ The PNPLA3 variant rs738409 disrupts normal triglyceride breakdown, leading to fat accumulation in the liver.^23^ Each PNPLA3 risk allele confers a 2.2‐fold increased risk of HCC, with GG homozygotes exhibiting up to a 5‐fold higher risk compared to CC individuals.^24^ The rs58542926 variant of TM6SF2 is associated with higher cholesterol and fatty acid production, with an increased risk of HCC by 1.92‐fold.^25^, ^26^ Similarly, MBOAT7, the rs641738 C>T variant, is associated with reduced hepatic MBOAT7 expression, leading to altered phospholipid composition, increased hepatic fat accumulation, and heightened ER stress.^27^ Moreover, the microsomal triglyceride transfer protein gene (MTTP) variant, rs745447480, has been identified as a cause of MASLD‐HCC in homozygous individuals.^28^ Additionally, the impairment of the glycolysis inhibitor GCKR via the rs1260326 (P446L) variant was found to be a key genetic modifier for MASLD to HCC progression, as recently described.^29^

Certain genes frequently mutated in MASH‐HCC include human telomerase reverse transcriptase (hTERT), tumor protein p53 (TP53), and catenin beta 1 (CTNNB1).^30^ Alterations in the hTERT gene allow cancer cells to maintain telomere length in HCC.^31^ Mutations in the Wnt/β‐catenin signaling pathway include gain‐of‐function in CTNNB1 and loss‐of‐function in AXIN1.^32^, ^33^ These mutations prevent β‐catenin from being properly degraded, which nuclear translocation sustains cell division, angiogenesis, tumor infiltration, and metastases.^34^

Finally, many metabolic and molecular alterations such as mutations in TERT, TP53, CTNNB1, and AXIN1 can be present across different etiologies. However, genetic variants like PNPLA3, TM6SF2, and MBOAT7 are more strongly linked to MASLD‐driven HCC, playing a role in other liver diseases. This underscores the complexity of metabolic regulation in liver cancer and the need for further studies to define MASLD‐specific mechanisms.

## 4 METABOLIC REPROGRAMMING IN MASLD‐HCC

**FIGURE 1:**
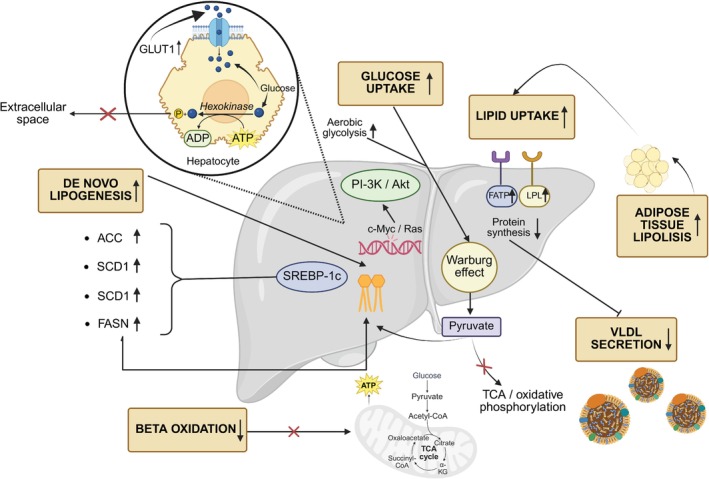
Metabolic reprogramming in HCC progression from MASLD. Metabolic reprogramming in cancer involves increased lipid uptake, de novo lipogenesis, adipose tissue lipolysis, decreased β‐oxidation, and VLDL secretion. Lipid synthesis is driven by the upregulation of SREBP1‐c, which enhances the expression of key enzymes like ACC, SCD1, ACLY, and FASN. Acetyl‐CoA and Malonyl‐CoA are FASN substrates. Lipid uptake is linked to increased FATP and LPL activity. Mutations in oncogenes like Ras and c‐Myc increase glucose uptake via PI‐3 K/Akt signaling, promoting GLUT1 expression and its translocation to the cell surface. Akt also enhances hexokinase activity, trapping glucose in the cell. The Warburg effect shifts metabolism toward gluconeogenesis and fat synthesis, diverting pyruvate away from the TCA cycle and oxidative phosphorylation.

Functional disruptions in metabolic pathways are underlying processes of MASLD‐HCC (Figure 1; principal pathways from current literature in Table 1).^35^, ^36^ Metabolic shift, via metabolic reprogramming, is driven by lipid uptake, de novo lipogenesis (DNL), adipose tissue lipolysis, decreased β‐oxidation, and very low‐density lipoprotein (VLDL) secretion.^37^, ^38^ IR hinders glucose oxidation and diverts carbohydrates into the DNL pathway, promoting cell proliferation.^39^ In MASLD and HCC, DNL is controlled by mammalian target of rapamycin (mTOR), ChREBP, and SREBP1‐c that, through acetyl‐CoA carboxylase (ACC), stearoyl‐CoA desaturase 1 (SCD1), and ATP citrate lyase (ACLY), sustain lipid synthesis.^40^, ^41^ Importantly, in MASLD‐HCC progression, a key driving factor is the uptake of circulating fatty acids, which is correlated with the increase in fatty acid transport proteins (FATPs) and hepatic lipases and lipoprotein lipase (LPLs) in response to IR.^42^ Moreover, patients with MASH have shown impaired synthesis and secretion of lipoproteins, resulting from compromised hepatocellular function.^43^ The reduced synthesis of apolipoprotein B‐100, essential for VLDL uptake, may be related to an imbalanced redox state, hyperinsulinemia, and lower protein levels in hepatocytes with consequent lipid accumulation in the liver. Aerobic glycolysis, commonly referred to as the Warburg effect, is a key feature of cancer‐related metabolic changes.^44^ Due to the Warburg effect, cells convert glucose into lactic acid and generate metabolic intermediates of lipid (e.g., glycerol‐3‐phosphate), proteins (e.g., serine), and nucleic acids synthesis (e.g., ribose‐5‐phosphate). Altered expression in oncogenes such as c‐Myc affects PI‐3/Akt signaling, promoting the expression of the glucose transporter 1 (GLUT1) and its relocation to the hepatocyte surface.^45^ While Myc activation is a recognized driver of glycolytic reprogramming in HCC, its prevalence varies by etiology. Myc is overexpressed in up to 70% of viral or alcohol‐related HCC, and functional studies show that Myc upregulates GLUT1 expression in HCC cell lines and tumor tissues.^46^ However, immunohistochemical analysis reveals that GLUT1 protein is detectable in approximately 13% of HCC cases, with higher expression correlating with advanced tumor stage and poor differentiation.^45^ These findings indicate that while Myc–GLUT1 axis activation is biologically significant, it is present in only a subset of HCCs, and its specific prevalence in MASLD‐HCC remains to be clarified. Further uptake of glucose is sustained by the Ras protein.^45^ Lack of insulin sensitivity promotes the gluconeogenic pathway and de novo fat synthesis, resulting in hyperglycemia and hepatic steatosis. Aberrant hyperactivation of glycolysis, due to the Warburg effect, is crucial in promoting the progression from MASLD to MASH, cirrhosis, and HCC.

**TABLE 1:** Relative importance and therapeutic promise of metabolic pathways in MASLD‐HCC.

Pathway	Role in MASLD‐HCC progression	Key targets/enzymes	Promising therapies/status	Relative importance
Lipid metabolism	Drives tumor growth, steatosis	SREBP‐1c, FASN, ACC, SCD1	FASN inhibitors (TVB‐2640), SREBP‐1c inh.	Highest
Glucose metabolism	Fuels rapid proliferation	GLUT1, HK2, mTOR	Metformin, HK2/GLUT1 inhibitors	High
Amino acid metabolism	Supports biosynthesis, redox balance	GLS1, glutamine transport	GLS1 inhibitors (CB‐839)	Moderate‐High
Mitochondrial metabolism	Provides metabolic flexibility	Complex I, IDH1/2	Metformin, IDH inhibitors	Moderate
Immune metabolism	Modulates immune escape	MCT1, PD‐1	MCT1 inhibitors + immunotherapy	Emerging

## 5 TWO‐WAY RELATIONSHIP BETWEEN EPIGENETICS AND METABOLIC REPROGRAMMING IN MASLD‐HCC

**FIGURE 2:**
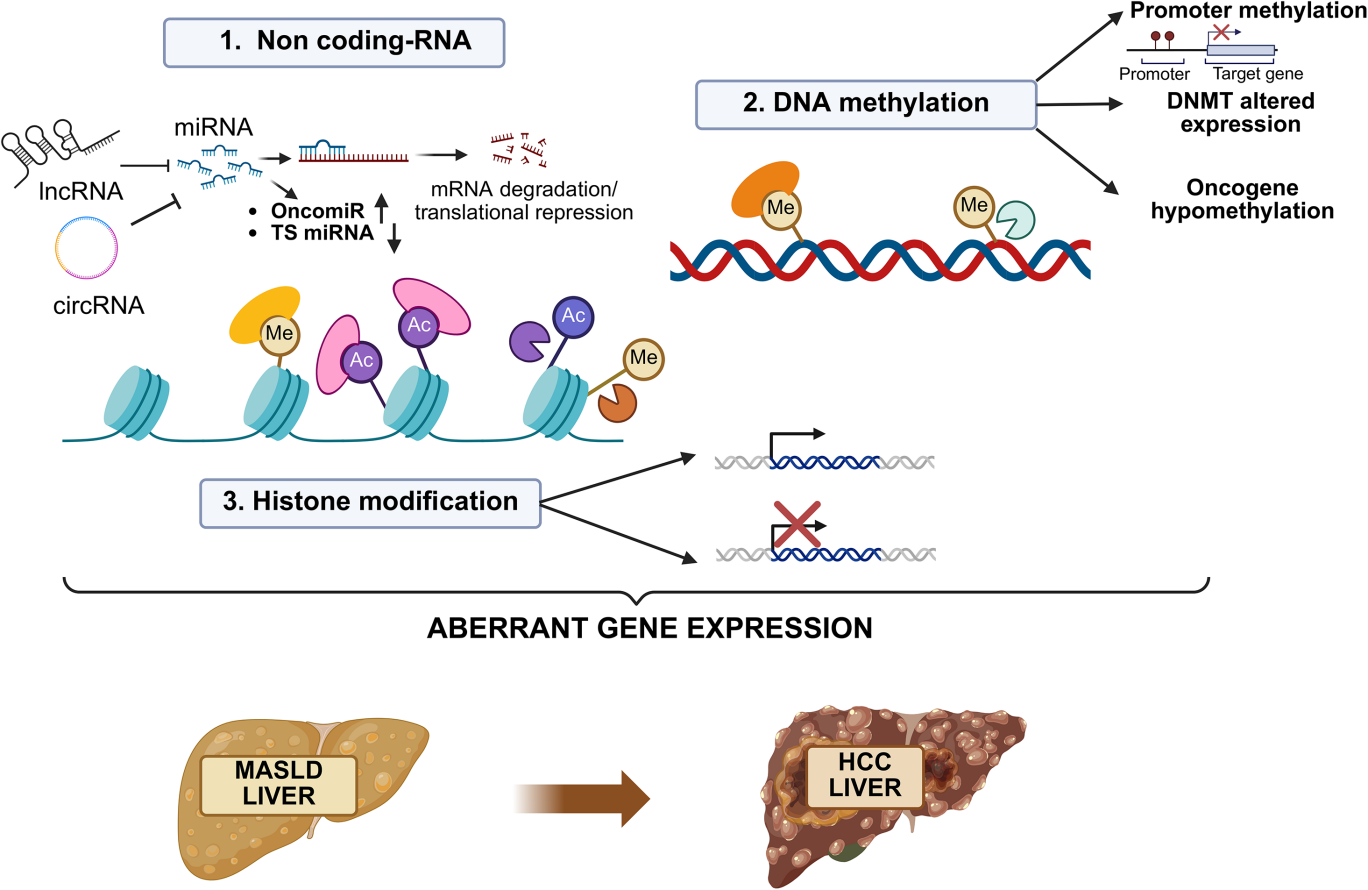
Principal epigenetic mechanisms involved in MASLD‐HCC progression. There are three regulatory systems: DNA methylation, chromatin remodeling through histone modifications, and non‐coding RNAs (ncRNA). In HCC, methylation changes include hypermethylation in tumor suppressor gene promoters, altered expression of DNMTs, and hypomethylation. Histones undergo key post‐translational modifications (PTMs) like methylation and acetylation. NcRNA are classified as long non‐coding RNA, microRNA, further divided into OncomiR and tumor suppressor (TS) miRNA, and circular RNA.

Emerging evidence highlights the strategic role of epigenetic changes in regulating the development and progression of MASLD and HCC.^7^ Epigenetic mechanisms are driven by extracellular factors and intracellular regulators (writers, erasers, and readers) like non‐coding RNAs (ncRNAs), DNA methylation, and histone modifications, as illustrated in Figure 2.

As previously described, tumors establish a two‐way communication between metabolic reprogramming and epigenetic alterations (Table 1).^7^ Indeed, various metabolic intermediates, including those ones influenced by dietary changes and obesity, reshape chromatin structure by regulating enzymes activity or directly modify chromatin, thereby affecting gene expression. Conversely, alterations in the expression or activity of specific epigenetic enzymes, such as DNA methyltransferases (DNMTs), histone acetyltransferases (e.g., p300/CBP), histone deacetylases (e.g., HDAC3, SIRT1), and histone methyltransferases (e.g., EZH2), can create a hepatic microenvironment that promotes MASLD progression and tumor development. This “positive MASLD microenvironment” it means the epigenetically and metabolically altered liver state that is conducive to disease progression, is characterized by increased lipid accumulation, inflammation, and fibrogenesis. These epigenetic changes not only drive neoplastic transformation by modulating fibrogenic, proliferative, angiogenic, and invasive pathways, but also disrupt normal metabolic regulation, further fueling carcinogenesis.

### 5.1 Non‐coding RNAs

Non‐coding RNAs (ncRNAs), including microRNAs (miRNAs), long non‐coding RNAs (lncRNAs), and circular RNAs (circRNAs), regulate gene expression and play a pivotal role in the progression from MASLD to HCC, as already described.^47^, ^48^ miRNAs are small single‐stranded RNAs (~22 nt) that modulate mRNA by degradation or translational inhibition, the dysregulation of which is involved in tumorigenesis, including HCC. For example, miR‐21 promotes fibrosis and hepatocarcinogenesis via phosphatase and tensin homolog (PTEN) downregulation and Kruppel‐like factor 5 (KLF5) suppression, which enhances liver cancer cell invasiveness and VEGF release.^49^, ^50^ Upregulation of miR‐155 in MASLD models reduces CCAAT enhancer binding protein beta (C/EBP‐β), contributing to tumor progression.^51^ Onco‐miRNAs like miR‐221, miR‐222, and miR‐103a promote cancer progression by inhibiting tumor suppressor genes.^52^ Conversely, tumor‐suppressive miRNAs, such as miR‐122 and miR‐26, are downregulated in HCC, leading to disease progression. For example, miR‐122 depletion impairs lipid metabolism and promotes fibrosis, but its restoration improves liver function in mice.^53^ miRNAs also influence metabolic dysregulation in MASLD‐HCC. For instance, miR‐21‐5p correlates with steatosis severity and alters the structure, function, and expression of proteins essential for mitochondrial integrity (e.g., as Drp1 and OPA1) that are regulated by PPAR‐α, altering hepatic metabolomics.^54^, ^55^

lncRNAs (>200 nt) contribute to HCC progression through tumorigenic pathways. MALAT1, upregulated in MASLD, enhances the Wnt/β‐catenin pathway, while NEAT1 promotes fibrosis and inflammation via the miR‐506/GLI3 axis.^56^, ^57^ Cancer susceptibility candidate 2 (CASC2) acts as a tumor suppressor by targeting miR‐155, restoring suppressors of cytokine signaling 1 (SOCS1) expression and inhibiting HCC proliferation.^58^ Nuclear enriched abundant transcript 1 (NEAT1) also modulates lipid metabolism, with its overexpression linked to increased ACC and fatty acid synthase (FASN) levels, promoting MASLD.^59^ NEAT1 is one of the most overexpressed long non‐coding RNAs in HCC. Multiple studies have shown NEAT1 upregulation in most HCC tissues compared to adjacent normal liver, with one cohort reporting high NEAT1 expression in 56 out of 81 HCC cases (69%).^60^ Elevated NEAT1 levels are associated with larger tumor size, higher grade, and poor prognosis.^61^, ^62^ While these data support a frequent role for NEAT1 in HCC biology, the exact proportion in MASLD‐HCC specifically is not yet established, highlighting the need for etiology‐stratified studies.

While most studies on circRNAs have focused on their role in HCC broadly, emerging data suggest that circRNA dysregulation may also contribute to MASLD progression and MASLD‐associated hepatocarcinogenesis. In fact, circular RNAs (circRNAs) are aberrantly expressed in HCC.^63^, ^64^ circRNA RHOT1 promotes HCC by promoting NR2f6 expression.^65^ circRNAs also influence fibrosis, as circCREBBP mitigates CCl4‐induced fibrosis by enhancing left–right determination factor 2 (LEFTY2) expression.^66^ Additionally, circMAT2B accelerates HCC progression by promoting glycolysis under hypoxic conditions, sponging miR‐338‐3p, and upregulating pyruvate kinase M1/2 (PKM2).^67^ However, direct evidence linking specific circRNAs to MASLD‐HCC remains limited, and further research is needed to clarify their disease‐specific roles.

### 5.2 Non‐coding RNA's role in oxidative lipid metabolism

Oxidative lipid metabolism is a key driver of hepatocyte injury and carcinogenesis in MASLD, as chronic lipid overload and increased β‐oxidation generate reactive oxygen species that promote inflammation, fibrosis, and malignant transformation. These mechanisms are particularly relevant in MASLD‐HCC, where metabolic stress and lipotoxicity accelerate disease progression. Recent literature on ncRNAs underscored that besides their role in metabolism, they can regulate cell death signaling like ferroptosis, which is inhibited by ncRNAs favoring cancer progression.^68^ For example, miR‐23a‐3p upregulation promotes resistance to sorafenib‐induced ferroptosis by directly binding acyl‐coenzyme A synthetase long chain family member 4 (ACSL4) mRNA in the 3’ UTR region.^69^ Another well‐described key regulatory system in ferroptosis is the solute carrier family 7 member 11 (SLC7A11)‐GSH‐GPX4 axis.^70^ GPX4, a glutathione (GSH) peroxidase, reduces lipid hydroperoxides using GSH, protecting cells from ferroptosis. Several dysregulated ncRNAs can boost this protective system in HCC, thereby increasing drug resistance.

### 5.3 Role of methylation in the metabolic reprogramming of MASLD‐induced HCC

DNA methylation, regulated by DNA methyltransferases (DNMTs), significantly influences gene expression by transferring methyl groups from S‐adenosylmethionine (SAM) to cytosine in CpG sites. DNMT1 maintains methylation patterns during cell division, while DNMT3A and DNMT3B perform de novo methylation. In cancer, methylation abnormalities include hypermethylation of tumor suppressor gene (TSG) promoters, altered DNMT expression, and hypomethylation of repetitive sequences.^71^

A study of 306 HCC patients revealed reduced overall DNA methylation with hypermethylation at TSG promoters like Ras association domain family member 1 (RASSF1) and neurogenic locus notch homolog protein 3 (NOTCH3), underscoring methylation's role in HCC development.^72^ Similarly, under MASH‐induced necroinflammation, hypomethylation leads to aberrant overexpression of oncogenes such as DDB1 and CUL4‐associated factor 4 like 2 (DCAF4L2) and tubulin alpha 1b (TUBA1B), contributing to carcinogenesis.^73^ Disheveled binding antagonist of beta catenin 2 (DACT2) is a tumor suppressor that inhibits Wnt/β‐catenin signaling. Aberrant activation of Wnt/β‐catenin is a shared feature of both advanced MASLD and HCC, promoting hepatocyte proliferation and tumor progression. In HCC, low DACT2 expression and Wnt signaling dysregulation are observed in 58.84% of HCC cases.^74^ Reduced DACT2 expression and increased β‐catenin signaling have also been reported in advanced metabolic liver disease, suggesting that epigenetic silencing of DACT2 may contribute to a mechanistic continuum from MASLD progression to HCC by permitting unchecked Wnt/β‐catenin activity.^75^

Epigenetic changes influence metabolic dysregulation in response to oxidative stress, fibrogenesis, and carcinogenesis. Hyper‐ or hypomethylation of specific genes, like PPARG coactivator 1 Alpha (PPARGC1‐A), affects fatty acid β‐oxidation and correlates with insulin resistance in MASLD patients.^76^ Studies also show methylation differences between mild and advanced MASLD, with hypomethylation‐linked upregulation of damage‐response genes in advanced disease. Recent research highlights mitochondrial DNA methylation's role in MASLD.^77^ Increased methylation, as shown in cells overexpressing methyltransferases, such as DNMTs and PEMT, can disrupt the expression of genes involved in mitochondrial function and bile acid metabolism. For example, PEMT‐mediated methylation is crucial for phosphatidylcholine synthesis, which is required for both VLDL secretion and bile acid production.^78^ Dysregulation of these methyltransferases may contribute to mitochondrial dysfunction and abnormalities in bile acid metabolism, promoting MASLD progression. These impairments, linked to bile acid metabolism abnormalities, contribute to MASLD/MASH progression and its transition to HCC.^79^

### 5.4 Histone modifications and histone variants

Histone modifications are key regulators of gene expression and chromatin structure, involving processes like lysine acetylation, methylation, and phosphorylation.

Acetyltransferases (HATs) activate gene expression, while deacetylases (HDACs) silence it. The coactivator p300, a member of the HAT family, regulates transcription factors like nuclear factor kappa B subunit 1 (NF‐κB) and carbohydrate‐responsive element‐binding protein (ChREBP), which are central to lipid synthesis and inflammation in hepatic steatosis and HCC.^80^ Overexpression of p300 in HCC promotes glycolysis‐related gene transcription through H3K27 and H3K18 acetylation, contributing to tumor progression and resistance.^81^

The expression of various HDACs is upregulated in MASLD and MASLD‐HCC and is associated with increased tumor aggressiveness, immune escape, and metabolic dysregulation.^7^ HDACs modulate chromatin accessibility and are divided into zinc‐dependent (HDAC1‐11) and NAD+‐dependent (sirtuin 1–7) classes.^82^ Dysregulation of HDACs is associated with increased tumor aggressiveness, insulin resistance, and HCC development, such as for HDAC8 or HDAC3.^83^, ^84^ Specifically, HDAC3 and HDAC8 have been implicated in promoting insulin resistance and β‐catenin activation in MASLD‐associated HCC, while inhibition of HDAC2 and HDAC3 has been shown to ameliorate hepatic steatosis and inflammation in experimental models. Moreover, HDAC activity in the liver correlates with metabolic parameters and disease severity in MASLD patients, highlighting the contribution of HDACs to the progression from steatosis to steatohepatitis and HCC.^85^ These findings underscore the relevance of HDACs as potential therapeutic targets in MASLD and its complications.

Sirtuins (SIRTs) play dual roles in cancer. SIRT1 suppresses tumor growth by reducing inflammation and promoting oxidative metabolism, while SIRT6's role is controversial, linked to both oncogenic effects and tumor invasion.^86^, ^87^

Histone methylation, mediated by enzymes like histone methyltransferases (HMTs) and histone lysine demethylases (KDMs), is critical for maintaining genome integrity. Modifications such as H3K27me3 (mediated by enhancer of zeste homolog 2 [EZH2]/KMT6) repress gene expression and enhance Wnt/β‐catenin signaling, driving HCC aggressiveness.^88^ EZH2 inhibitors have shown potential in boosting immune‐mediated cancer cell eradication.^89^ Epigenetic modifications also influence cancer stem cell (CSC) metabolism in HCC. Loss of the histone variant macroH2A1 in poorly differentiated tumors leads to CSC‐like characteristics and triglyceride accumulation.^90^

Furthermore, emerging histone post‐translational modifications (HPTMs) have been recently described as having an effect on HCC biology, including tumor proliferation, metabolic reprogramming, immune evasion, and therapeutic resistance.^91^ Among these, histone lactylation, directly linking cellular metabolism to epigenetic regulation, has emerged as a critical player. For example, lactate‐driven histone H2B lactylation has been shown to promote senescence resistance in HCC by modulating the expression of target genes like NDRG1, thereby contributing to tumor progression and heterogeneity.^92^ Furthermore, histone serotonylation represents another layer of this complex regulatory network. The enzymatic activity of Transglutaminase 2 (TGM2) mediates histone H3 glutamine 5 serotonylation (H3Q5ser), a modification that actively promotes HCC progression. This occurs, at least in part, through the potentiation of the MYC oncogenic signaling pathway, identifying TGM2‐mediated serotonylation as a novel axis in HCC pathogenesis and a potential therapeutic target.^93^ The continued elucidation of these novel HPTMs, their specific writers, readers, and erasers, and their functional consequences in HCC is crucial. However, the role of HPTMs in MASLD‐associated HCC has not been fully addressed, and more studies are needed to further characterize the HPTMs in this specific context.

### 5.5 Role of epi‐transcriptomic modifications in metabolic reprogramming of HCC

Epi‐transcriptome is also involved in the metabolic reprogramming of HCC. N6‐methyladenosine (m6A) methylations are internal mRNA modifications catalyzed by m6A writer methyltransferases such as methyltransferase 3, N6‐Adenosine‐methyltransferase complex catalytic subunit (METTL3) and removed by eraser demethylases like FTO and AlkB homolog 5 (ALKBH5).^94^ Analysis of the m6A methylome in mice with a high‐fat diet indicated significant hypermethylation in lipid metabolism‐associated mRNA genes.^95^ This alteration exacerbates hepatic FA synthesis and storage, contributing to the advancement of MASLD and HCC.^96^ Specifically, Yang et al. used a MASLD mouse model knockout for Tmem30a, which is based on lipid metabolism disorder caused by type 2 diabetes mellitus (DM2) and not simply attributable to SREBP1‐c hyperactivation. In this case, MASLD is linked to excessive m6A methylation in ACLY and SCD1 mRNAs. The same study also demonstrated that the alteration in lipid metabolism is correlated with increased m6A methylation of genes involved in DNL by the upregulated expression of METTL14 and METTL3 in HCC patients.

### 5.6 Mechanisms through which metabolic reprogramming influences epigenetic modifications in HCC

**FIGURE 3:**
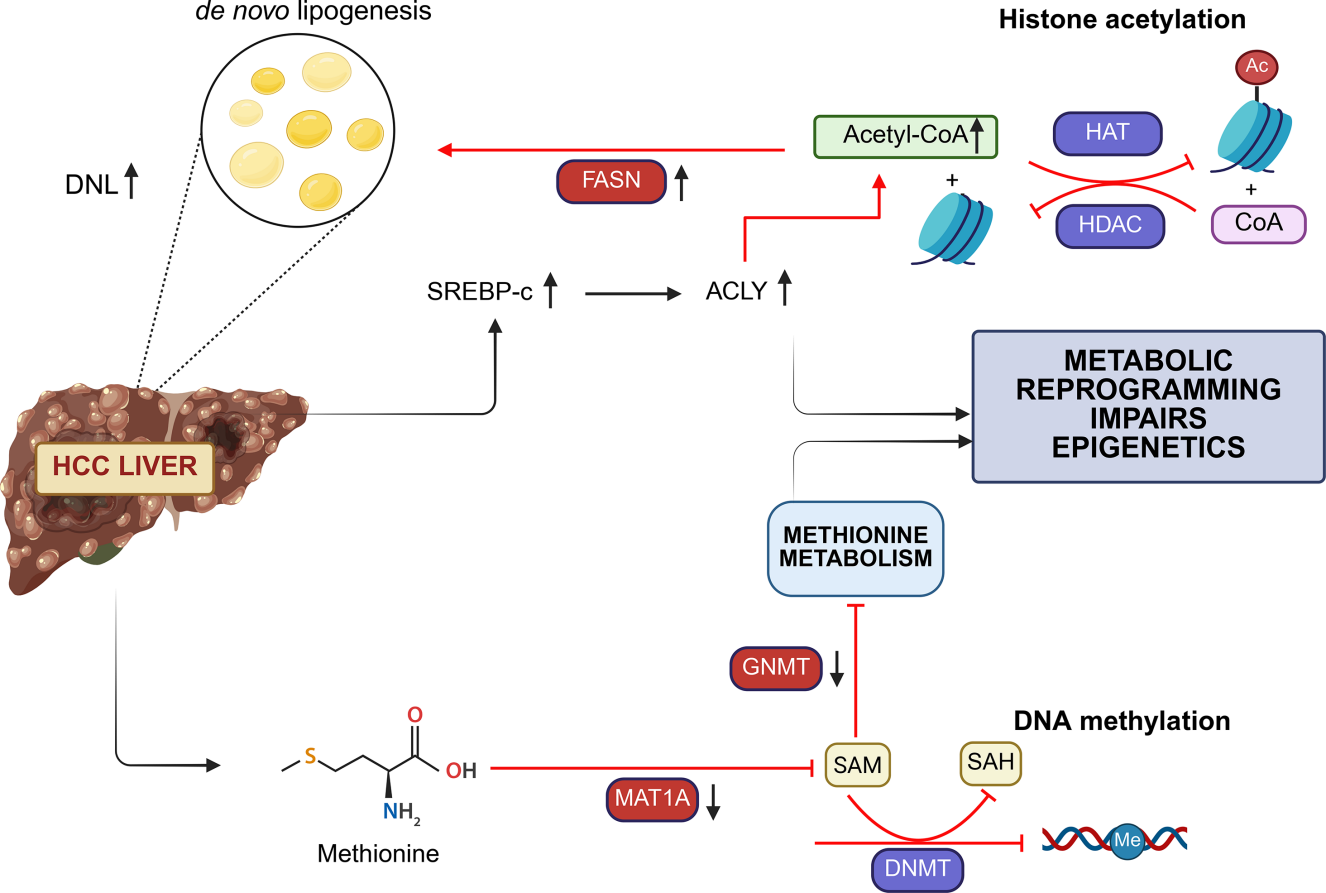
Mechanisms through which metabolic reprogramming influences epigenetics in MASLD‐HCC. Hyperactivated FASN drives Acetyl‐CoA into lipid synthesis, disrupting HAT/HDAC activity. Deficiency in DNMT or MAT1A, or both impacts SAM/SAH balance that is essential for the methylation activity by epigenetic modifiers, leading to epigenetic changes in metabolic and carcinogenic pathways.

Histone acetylation, driven by acetyl‐CoA derived from citrate, links glucose metabolism to epigenetic regulation. Disruptions in HAT/HDAC activity impact gene expression, contributing to MASLD‐associated cancer development.^97^ SIRT2, overexpressed in HCC, deacetylates phosphoglycerate mutase 5 (PGAM5) at lysine K191, activating its phosphatase function toward NADP‐dependent malic enzyme (ME1), which promotes lipid synthesis and tumor cell proliferation.^98^ ME1 connects glycolysis to the TCA cycle, further driving fatty acid biosynthesis.^98^ S‐adenosylmethionine (SAM) is a critical methyl group donor in methionine metabolism, and its imbalance disrupts epigenetic regulation, contributing to HCC progression.^99^, ^100^, ^101^, ^102^ Glycine‐N‐methyltransferase (GNMT), the primary hepatic methyltransferase, regulates SAM availability and HAT activity.^103^ GNMT deficiency elevates SAM levels, leading to MASLD and HCC by promoting metabolic shifts toward polyamine catabolism, lipogenesis, and GSH synthesis.^99^ Methionine adenosyltransferase 1A (MAT1A), essential for SAM synthesis, also plays a crucial role in SAM/SAH balance. MAT1A deficiency lowers SAM levels and predisposes individuals to MASLD‐associated HCC, while MAT1 promoter hypermethylation has been linked to hyperhomocysteinemia in cirrhosis.^104^, ^105^ Both endogenous SAM imbalance and nutritional methyl group deficiency impact epigenetic regulation. In murine models with a methyl‐deficient diet, hepatic steatosis leads to significant demethylation of the genome and repetitive elements due to reduced DNMT1 and KMT RIZ1 activity, accompanied by the loss of H3K27 and H4K20 trimethylation, impairing genome integrity.^106^ Main mechanisms of metabolic reprogramming influencing epigenetics in MASLD‐HCC are in Figure 3.

## 6 GUT MICROBIOTA AND EPIGENETIC ALTERATIONS IN METABOLIC REPROGRAMMING OF MASLD‐INDUCED HCC

Intestinal dysbiosis results in the production of metabolites including short‐chain fatty acids (SCFAs), bile acids (BAs), ethanol, tryptophan metabolic intermediates, and branched‐chain amino acids, which are associated with MASLD and HCC development.^107^ These metabolites actively take part in key epigenetic alterations in HCC development. One significant modification is the alteration of DNA methylation and acetylation by SCFAs. SCFAs reduce the expression of DNMT1 and methyl‐CpG binding domain protein 2 (MBD2), which prevents these enzymes from binding to the promoters of adiponectin and resistin, two important adipokines in white adipose tissue.^108^ The mRNA expression of adiponectin and resistin is upregulated due to promoter gene demethylation, thereby restoring gene transcription. Adiponectin and resistin can influence MASLD progression. SCFAs also deregulate the balance between acetylation and deacetylation by increasing HATs' activity. SCFAs acetylate p70S6 kinase, which is normally deacetylated in T cells, thereby promoting T cell differentiation in inflamed liver tissue.^108^ Conversely, emerging evidence suggests that acetate produced by normal microbiota has protective functions. An interesting study revealed that the immunomodulatory power of bacterial‐derived acetate on innate lymphoid cells ILC3 involves an interesting epigenetic mechanism. In mice with HCC, where *Lactobacillus reuteri* is significantly reduced and ILC3‐producing interleukin (IL)‐17A is increased, fecal transplantation and acetate administration decrease tumor growth and improve tumoral sensitivity to programmed cell death protein 1 (PD‐1) treatment.^109^ Finally, gut microbiota alterations across the MASLD spectrum, with obesity and disease severity (fibrosis, HCC), drive distinct microbial signatures. Lean MASLD and MASLD‐HCC patients show unique microbial and mycobiome features, though more research is needed for lean MASLD (Table 2).

**TABLE 2:** Key microbiota features.

Group	Key microbiota features	Notes
Lean MASLD	Distinct mycobiome changes (↑ *Mucor*, *C. albicans*), (low data)	Microbial shifts independent of obesity^125^
Obese MASLD	↑ Translocation of gut bacteria (e.g., *Enterococcus*, Morganellaceae), ↑ Bacteroidetes/Firmicutes (liver), ↓ Lachnospiraceae	Obesity drives fecal microbiome more than MASLD^126^
MASLD‐HCC	↑ Pathogenic bacteria (*Bacteroides*, *E. coli*, Enterobacteriaceae), ↓ diversity, ↑ fungal taxa (*C. albicans*)	Linked to advanced fibrosis, barrier dysfunction^127^

## 7 TARGETING EPIGENETICS AND METABOLIC REPROGRAMMING OF HCC

Recent years have been characterized by significant advances in the clinical development of therapies targeting epigenetic mechanisms and metabolic pathways in MASLD‐HCC. However, most are still in early‐phase trials or preclinical stages. Several classes of epigenetic drugs (“epidrugs”), including DNA methyltransferase inhibitors (DNMTis), histone deacetylase inhibitors (HDACis), and bromodomain inhibitors, are under investigation for HCC, with some showing promise specifically in MASLD‐driven disease (Table 3). These epidrugs, which specifically target epigenetic regulators, offer potential long‐term reprogramming and restoration of chemotherapy sensitivity by modifying the tumor microenvironment (TME).^110^, ^111^ DNMT inhibitors like 5‐azacytidine and decitabine restore tumor suppressor gene expression, enhance differentiation, and improve sorafenib sensitivity.^112^ The second‐generation DNMT inhibitor guadecitabine is more resistant to enzymatic degradation. HDAC inhibitors such as panobinostat induce apoptosis, suppress angiogenesis, and modulate tumor metabolism.^113^ Among histone‐modifying drugs, EZH2 inhibitors like GSK126 enhance natural killer (NK) cell activity in HCC, while dual inhibitors like CM272 (targeting DNMT1 and G9a) improve tumor suppressor gene activation while reducing tumor proliferation.^89^, ^114^ Epigenetic modifications are also being explored for MASLD‐HCC chemoprevention, as evidenced by bromodomain and extra terminal domain 4 (BRD4) inhibitors, which significantly reduced tumor progression in MASH‐HCC models.^115^

**TABLE 3:** List of clinical trials.

Target class	Drug	Mechanism of action	Development stage
DNMT inhibitors	5‐Azacytidine, Decitabine	Restore tumor suppressor gene expression, enhance differentiation, improve sorafenib sensitivity^113^	Clinical trials
	Guadecitabine	Second‐generation, more resistant to enzymatic degradation^113^	Clinical trials
HDAC inhibitors	Panobinostat	Induces apoptosis, suppresses angiogenesis, modulates tumor metabolism^114^	Clinical trials
EZH2 inhibitors	GSK126	Enhances NK activity^115^	Preclinical
Dual DNMT1/G9a inhibitors	CM272	Activates tumor suppressor genes, reduces tumor proliferation^90^, ^115^	Preclinical
BRD4 inhibitors	(Not specified)	Reduced tumor progression in MASH‐HCC models^113^	Preclinical
miRNA targeting	Anti‐miR‐221/222	Suppress anti‐apoptotic signaling via Bmf modulation^117^, ^118^, ^119^	Preclinical
Glycolysis (HK2)	Chrysin	Inhibits HK2, reduces glycolysis, induces mitochondrial apoptosis in dose‐dependent manner^121^	Preclinical
Lipogenesis (FASN)	FASN inhibitors	Block lipid anabolism in HCC^122^, ^123^	Preclinical
p300/CBP inhibitors	A‐485	Selective acetylation inhibitor, anti‐tumor effects in lineage‐specific cancers^124^	Clinical trials (other cancers)
	B029‐2	Inhibits acetylation activity, reduces glycolysis and metabolic gene expression^81^	Preclinical

Inhibiting oncogenic miRNAs provides another potential epigenetic intervention. miR‐221/222, which suppress apoptosis by modulating Bcl2‐modifying factor (Bmf), is a key target.^116^ Antisense oligonucleotides (antagomirs) effectively silence miRNAs, with studies showing miR‐221 antagonists markedly reduce expression in vivo.^117^, ^118^

Emerging evidence supports targeting ferroptosis against drug resistance.^119^ While ncRNAs are known to regulate ferroptosis through lipid and iron metabolism modulation, no inhibitor targeting the ncRNA‐ferroptosis axis has reached clinical trials.

Since metabolic dysregulation is central to MASLD‐HCC, targeting key metabolic pathways has shown therapeutic potential. Hexokinases (HKs) catalyze the first irreversible step of glycolysis, making them attractive targets. In HCC models, chrysin, a natural flavone, inhibits HK2, reducing glycolysis and inducing apoptosis by activating mitochondrial Bax in a dose‐dependent manner.^120^ Dysregulated lipid anabolism is another promising target, particularly fatty acid synthase inhibitors.^121^, ^122^

Inhibiting p300/CBP has shown efficacy in hematologic malignancies, prostate, and colorectal cancer. The selective p300/CBP inhibitor A‐485 exhibited anti‐tumor effects in lineage‐specific cancers.^123^ Recent findings by Cai et al. further highlight p300/CBP's role in regulating glycolysis, amino acid metabolism, and nucleotide synthesis. The B029‐2 inhibitor reduces glycolysis and metabolic gene expression by blocking p300/CBP acetylation activity.^81^

### 7.1 Challenges of targeting epigenetic modifications

Besides the exciting therapeutic opportunities, significant challenges limit their clinical application, particularly regarding drug specificity and off‐target effects. The lack of specificity increases the risk of adverse effects, especially in solid tumors like HCC, where the tumor and surrounding healthy tissue may both be affected. In fact, systemic administration can expose non‐diseased tissues. DNA methyltransferase (DNMT) and histone deacetylase (HDAC) inhibitors lack locus specificity and act broadly across the genome, leading to widespread alterations in gene expression that can disrupt normal cellular functions and cause unintended toxicity.^124^ Moreover, the complex and heterogeneous nature of HCC further complicates the identification of optimal epigenetic targets, as different etiologies and tumor subtypes may be characterized by distinct epigenetic landscapes. While novel approaches such as locus‐specific epigenetic editing and RNA‐based therapies are under development to improve precision, these remain largely experimental and face additional challenges related to delivery, durability, and safety before clinical translation. Therefore, although the reversibility of epigenetic changes makes them attractive therapeutic targets, overcoming the issues of specificity, off‐target effects, and efficient delivery is essential for the successful application of epigenetic therapies in MASLD‐HCC.

## 8 CONCLUSIONS AND FUTURE PERSPECTIVES

Global burden of MASLD‐associated HCC is increasing, with early diagnosis as a major challenge; late‐stage detection drives poor prognoses. Sorafenib remains a frontline therapy. Rising resistance rates underscore the urgent need for alternative treatment strategies. Emerging evidence highlights the crucial interplay between metabolic reprogramming and epigenetic alterations in MASLD‐HCC progression, revealing promising therapeutic approaches such as targeting lipid metabolism, ferroptosis pathways, and epigenetic regulators, including DNA methylation, histone modifications, and non‐coding RNAs. Although epidrugs and metabolic inhibitors have shown efficacy in preclinical models, clinical translation remains limited, with experience from other cancer types indicating that epigenetic therapies often require combination approaches for sustained benefit.

To bridge these gaps, future research in MASLD‐HCC may prioritize the development and validation of etiology‐specific biomarkers and risk stratification tools to enable earlier detection, particularly in non‐cirrhotic patients. Mechanistic studies integrating the latest tools are needed to unravel the unique metabolic and epigenetic interplay in MASLD‐driven hepatocarcinogenesis and to identify novel druggable targets and patient subgroups most likely to benefit from targeted interventions. Clinical trials may enroll and stratify specifically MASLD‐HCC patients, rigorously evaluating existing and novel agents, including metabolic modulators either as monotherapies or in combinations. Moreover, given the complex tumor microenvironment and potential for immune evasion in MASLD‐HCC, the combination of strategies aimed at integrating metabolic or epigenetic agents with immunotherapies, such as immune checkpoint inhibitors, may be promising. Furthermore, research should address the optimal sequencing of therapies and the development of predictive biomarkers for response and resistance. Importantly, continued refinement of lifestyle and preventive interventions remains essential, as these may synergize with pharmacologic approaches to reduce the overall burden of MASLD‐HCC.

## AUTHOR CONTRIBUTIONS

**Chiara Aiello:** Conceptualization; writing – original draft; writing – review and editing; visualization. **Eric Felli:** Conceptualization; writing – original draft; writing – review and editing. **Teresa Musarra:** Supervision. **Lorenzo Nevi:** Writing – review and editing; visualization. **Annamaria Altomare:** Supervision. **Jordi Gracia‐Sancho:** Writing – review and editing; supervision. **Andrea Baiocchini:** Conceptualization; supervision. **Simone Carotti:** Conceptualization; writing – original draft; writing – review and editing; supervision.

## CONFLICT OF INTEREST STATEMENT

The authors declare no conflicts of interest.
